# Economic evaluation of Learning Through Play Plus in comparison to usual care for depressed mothers alongside a randomised controlled trial

**DOI:** 10.1186/s12913-026-14113-0

**Published:** 2026-02-04

**Authors:** Tehmina Ashraf, Mohsin H. Alvi, Akbar Ullah, Tayyeba Kiran, Anil Gumber, Siham Sikander, Nasim Chaudhry, Imran B. Chaudhry, Nusrat Husain

**Affiliations:** 1 Pakistan Institute of Living and Learning, Karachi, Pakistan; 2 School of Health Sciences, The University of Manchester, 176 Oxford Rd, Manchester, M13 9PY UK; 3 Health Services Academy, Islamabad, Pakistan; 4 Sheffield Hallam University, Sheffield, UK; 5 University of Liverpool, Liverpool, UK; 6 Ziauddin University, Karachi, Pakistan; 7 Global Centre for Research on Mental Health Inequalities , Mersey Care NHS Foundation Trust, Prescot, UK

**Keywords:** Economic evaluation, Depression, Cost-effectiveness analysis, Cognitive behavioural therapy, Randomised controlled trial, Parenting intervention

## Abstract

**Background:**

Research on the cost-effectiveness of postnatal depression treatments is limited in developing countries and among ethnic minorities in developed nations. This study presents a health economic evaluation of an integrated parenting intervention, Learning Through Play Plus (LTP+), for postnatal depression and child development, compared to treatment as usual (TAU), alongside a randomised controlled trial in Pakistan.

**Methods:**

Using data on 764 mothers from the ROSHNI-PK trial, we conducted an economic evaluation over a six-month time horizon to assess the cost-effectiveness of LTP+ from the perspective of health, social care and patient in Pakistan. Cost-utility was analysed using EQ-5D-3L instrument while cost-effectiveness was assessed using the Edinburgh Postnatal Depression Scale (EPDS) for mother and the Ages and Stages Questionnaires: Social-Emotional (ASQ: SE) for the child. Cost-utility analysis was conducted for mother-only and partially for mother-child dyad, as EQ-5D-3L data were collected for mother only, whereas cost-effectiveness was conducted for both dyad and mother-only. Incremental cost-effectiveness ratios (ICERs) were calculated from adjusted mean costs and outcomes.

**Results:**

Delivering LTP+ cost US $68.7 per dyad. LTP+ increased maternal costs by $33 (95% CI: $24: $43) and gained 0.06 (CI: 0.05: 0.07) quality-adjusted life-years (QALYs) compared to TAU-only. For the dyad, costs increased by $15 (CI: $4: $25). The ICER per maternal QALY gained was $582 (CI: $404: $769) when only maternal costs were considered, and $258 (CI: $75: $442) when dyad costs were considered. Dyad recovery (normal EPDS and ASQ: SE scores) cost $29 (CI: $11: $49), while maternal recovery alone cost $80 (CI: $53: $111). Dyad analyses showed that LTP+ has a 100% likelihood of being more cost-effective than TAU-only at willingness-to-pay thresholds of $65 per recovery or $600 per QALY gain. Analyses with varying combinations of LTP+ and healthcare costs and outcomes confirmed that the cost per QALY gained from LTP+ consistently remained below Pakistan’s annual per capita gross domestic product (GDP).

**Conclusion:**

LTP+ combined with TAU resulted in higher QALYs and recovery rates but at higher costs than TAU alone. While not cost-saving, LTP+ has a very high likelihood of being more cost-effective than TAU alone if the willingness-to-pay per QALY is at least 25% of Pakistan’s 2015 annual GDP per capita.

**Trial:**

# NCT02047357; Pre participant trial enrolment, 21/01/2014

**Supplementary Information:**

The online version contains supplementary material available at 10.1186/s12913-026-14113-0.

## Background

Worldwide, an estimated 17.2% of postnatal women (i.e., those in the first year after childbirth) experience a mental health disorder called postnatal or postpartum depression [1]. Postnatal depression prevalence is higher in developing countries than in the developed world [2]. Its prevalence is around 39.9% in South Africa and 22.32% in South Asia [1].

Depression is an important public health problem as it has adverse effects on quality of life and can cause disability and suicide in women, besides its economic impacts in the form of higher unemployment, lower productivity and income, and higher healthcare expenditure among affected households [3–5]. Depression among mothers increases the probability of poor child outcomes such as cognitive and socio-emotional problems, impaired physical health, and poor performance in schools [6–8]. Antidepressants are clinically effective, but new mothers may be reluctant to use them due to concerns about side effects for themselves and their children, as well as fears of long-term dependence [9, 10]. Therefore, cognitive-behavioural therapy (CBT) is recommended as a first-line treatment and has proven effective for postnatal depression [9, 10].

Many mothers with postnatal depression in low- and middle-income countries remain untreated due to ill-equipped health systems, lack of trained professionals, poverty, illiteracy, lack of awareness, fear of stigma, and societal myths [11–13]. Patients or their families often do not recognise postnatal depression as an illness, and husbands typically make healthcare decisions for their wives. Consequently, women are often not allowed to visit city hospitals and seek local treatments instead [13]. The standard care, referred to as treatment as usual (TAU) in this study, predominantly includes routine follow-ups by Lady Health Workers who provide primary maternal and child healthcare but have no specialist mental health training. Mental healthcare often involves seeking help from traditional healers, with little reliance on formal mental health services [11–15].

Despite the widespread prevalence, a recent systematic review that reviewed the cost-effectiveness of all pharmacological and non-pharmacological treatments for depression in lower- and middle-income countries (covering the period 2000 to December 2022) only identified three trial based intervention for depression [16].

A low-intensity intervention, the Thinking Healthy Programme (THP), was tested to treat perinatal depression (i.e., depression during pregnancy or in the first year after childbirth) among women in Pakistan [11]. Two subsequent studies examined the efficacy and cost-effectiveness of peer-delivered THP in India and Pakistan, where the peers were laywomen from the community with no prior health training or experience but shared sociodemographic and life experiences with the target population [17, 18]. In Pakistan, the peer-delivered THP showed no significant impact on perinatal depression (measured by Patient Health Questionnaire-9 (PHQ-9) scores) between the treatment and control groups, even after 18 group-based booster sessions from the 6th to the 36th month postnatally [19]. The intervention cost $236 (2015 US dollars) per additional recovery from depression at the 6th month postnatally under the health system perspective and $215 per additional recovery under the societal perspective [17]. In India, while its clinical effectiveness was weak, the peer-delivered THP was found to be cost-effective [18].

The Learning Through Play Plus (LTP+) intervention adapted the THP for group settings and combined it with the Learning Through Play (LTP) program, creating an integrated intervention for postnatal depression and child socio-emotional development. Considering the cultural barriers, the intervention was culturally adapted and delivered at participants’ homes or in the nearest basic health units. Unlike peer-delivered THP [17], both LTP+ and the original THP were found to be highly effective in treating depression when delivered by trained Community Health Workers (CHWs) [11, 14]. CHWs are healthcare workers, typically community members, who are trusted and respected and serve as a link between people’s homes and formal government primary healthcare clinics. However, unlike peers, they do not have lived experience of depression [14, 20]. For LTP+ delivery, they were trained in the relevant content and regularly supervised by trained research psychologists [14]. However, the question of whether LTP+ or THP is cost-effective when delivered by CHWs is not addressed in earlier studies. Therefore, we conducted a health economic evaluation over a 6-month time horizon to compare the cost-effectiveness of LTP+ added to TAU with TAU-only, alongside a randomised controlled trial, from a broader perspective encompassing both public, social and family-related healthcare costs in Pakistan.

## Methods

### Study design and population

The Roshni Trial (Trial # NCT02047357) was a two-arms Cluster Randomised Control Trial (CRCT), and ethical approval for the trial was obtained from the ethics committee of Karachi Medical and Dental College (Ref #0019/13). Details of the trial and its clinical results are published elsewhere [14]. Briefly, the trial was conducted in Gadap town, Karachi, between January 2014 and December 2015. A cluster of 120 villages was randomised (with villages as the unit) into either the LTP+ plus TAU arm or the TAU-only arm, in a 1:1 ratio. Clinically diagnosed depressed mothers aging 18–44 years, having 0–30 months old children were recruited. Mothers diagnosed with a major depressive episode (DSM-IV 4th Edition) and scoring > 12 on the Edinburgh Postnatal Depression Scale (EPDS) were eligible for this study. Participants diagnosed with serious physical illnesses, those who were not permanent residents of the catchment area, those with active suicidal ideation or the presence of any other severe mental disorder were excluded from the study. Mothers were recruited by CHWs either from their homes or from basic health units. Written informed consent was obtained from all patients. Trained researchers, blinded to treatment allocation, collected data at baseline and at 3 and 6 months post-baseline. Further details about the study design, procedures, and participants are available in the published work [14].

Methods and analyses for the economic evaluation adhere to CHEERS reporting guidelines and previous economic evaluation papers [21–24]. All procedures comply with the ethical standards of the relevant national and institutional human experimentation committees and the Helsinki Declaration.

### Treatment as usual

TAU was available to participants in both trial arms and consisted of routine follow-ups by Lady Health Workers. These workers, women having at least eight years of education, operate under government-run programmes to deliver primary healthcare and address unmet needs in rural and urban slum areas, often within their own communities [15]. They receive standardised pre- and in-service training in basic and primary healthcare, conducted by physicians and other healthcare professionals [14, 15]. Their responsibilities include maternal and child healthcare, such as family planning, antenatal and postnatal care, breastfeeding, nutrition, hygiene, vaccination promotion, polio surveillance, and basic health education (see Folz & Ali [15] for further details).

### Intervention

LTP+ comprised 10 group sessions integrating child development techniques and CBT. LTP included parental information in pictorial form while the CBT was based on THP [11, 14]. The LTP+ intervention contains strategies to promote child development in five areas (sense of self, physical development, relationships, understanding of the world, and communication) and helps mothers to identify and change their unhelpful thoughts related to their own health and wellbeing, their child’s growth and development, and their relationships. The sessions were delivered by trained CHWs over a period of 3 months, with facilitation from master’s level LTP+ trained psychologists. Each session lasted between 60 and 90 min and was delivered at the home of one of the group participants. CHWs attended training sessions of 18 h, and monthly training refreshers during the intervention. Two researchers attended the sessions as delegates to evaluate fidelity. All the participants were assessed at baseline, 3 months (end of intervention), and 6 months (post-baseline). The outcome assessments were done by blind raters.

### Resource use and unit costs

The economic evaluation adopted a health, social care, and patient-family perspective, incorporating the costs of delivering the LTP+ intervention and mother and child healthcare resource use (to both the health system and participants). Pakistan has a parallel existence of public and private healthcare services, besides homeopathic clinics and religious healers (Imams or Hakims), attracting patients with different societal myths. The public services provide free consultation, medical diagnostics, inpatient beds, and a limited number of free medications in cases of emergency attendances/admissions. The private medical, homeopathic, and most of the religious healers’ services need out of pocket payment. The homeopathic clinics and Hakims charge a single payment covering their service fee and the medication they provide. The religious scholars either provide free services or charge a single payment for their religious procedures.

Keeping this in view, two questionnaires were used to collect healthcare use data at the 3- and 6-month follow-ups, each covering healthcare use in the preceding three months. A Client Service Receipt Inventory recorded numbers of inpatient hospital days, outpatient hospital visits, emergency attendances, and visits to general practices, as well as visits to traditional healers (homeopathic clinics, Imams or Hakims, and other services). Though privately owned, data on traditional healing services were collected separately from private medical services, as most people do not consider these services as medical healthcare. Since these services charge a single payment covering their service fee and medication, the number of visits and the unit cost helped us determine the total cost incurred on these services.

To cover the amount of out-of-pocket money spent on private medical services or paid during visits to public health services, a second self-administered bespoke tool asked each mother about the number of visits to doctors for both their own and their child’s medical care during the last 3 months, as well as the total out of pocket expenses incurred. This questionnaire was delivered as part of a socio-demographic survey and did not request further details about the types of healthcare services accessed or the specific health conditions for which these services were used.

For hospital and general practice, unit cost data were collected from the four hospitals operating in the catchment area (Baqai Hospital Gadap, Pattel Hospital Gadap, Gadap RHC, and SASSI Clinic). Each hospital provided data on the minimum and maximum expenditures incurred for each type of service provision. Unit cost data for traditional treatments were collected from three familiar sites operating in the area (Raeesh Jokhio, Fida Husain Jokhio, Rahim Bux). The unit cost of each type of healthcare resource use was calculated by averaging the information across the different cites (See additional file, Table A1). All costs, including the LTP+ delivery cost (Table 1), unit costs (additional file Table A1), and healthcare use costs (additional file Table A2), are reported in 2015 Pakistani rupees (Rs) to avoid overly small figures when converted to United States (US) dollars. For the analyses, costs were converted to US dollars ($) at the end-year (2015) International Monetary Fund exchange rate (1 $ = 105 Rs), consistent with previous cost-effectiveness studies for the same year [17, 25].

LTP+ costs included therapist training costs, transportation and travel costs, payments made to therapists (CHWs) for session delivery, development and printing of manuals, babysitters, and venue charges. Each mother allocated to the treatment arm received Rs. 300 for the time they allocated to participate in LTP+ sessions. Since the health and social care sectors do not make such payments in practice, the Rs. 300 paid to each participant in the LTP+ group was excluded from the base-case analysis [26]. Intervention costs, sourced from trial records, were applied to mother costs as in Cox et al. [24]. Following standard practice, the base-case economic evaluation includes only LTP+ delivery costs (referred to as recurrent costs in Table 1), excluding setup costs such as manual development, research-related expenses, or training of CHWs, as these are not required for subsequent implementation [27]. The impact of including setup (non-recurrent) costs is explored in the sensitivity analyses.

Total costs for mothers and dyads were calculated by multiplying the number of each healthcare resource used during the trial period by their respective unit costs and summing the results. Costs were assessed from the perspective of health, social care, and the family, as reliable societal cost data were not available. For children, only out-of-pocket healthcare expenses were collected, limiting the dyad analyses to partial healthcare use data for the child.

### Outcomes measures

For the cost-utility analysis, the health benefit measure was the mother’s health-related quality of life. For the cost-effectiveness analysis, the measure was the mother’s recovery from postnatal depression, while the dyad analysis considered both the mother’s recovery and the child’s achievement of normal socio-emotional wellbeing scores. Health-related quality of life data were collected through the EQ-5D-3L instrument, administered at the baseline and each follow-up [28]. The newly published Pakistani tariff was used for the conversion of EQ-5D-3L health states to health utility scores [29]. Quality-adjusted life years (QALYs) were calculated using the area under curve calculation [30]. Postnatal depression was assessed using the Edinburgh Postnatal Depression Scale (EPDS) [31], with recovery defined as an EPDS score of 10 or less [32]. Child socio-emotional development was measured using the Ages and Stages Questionnaires: Social-Emotional (ASQ: SE), which identifies potential social-emotional challenges and supports early intervention [33]. Higher total scores indicate more severe concerns, with each age-specific questionnaire having its own established cut-off score [34]. A score below the age specific cut-off was assigned a value of one, indicating normal socio-emotional wellbeing.^1^ The dyad recovery indicator was assigned a value of one when both mother and child achieved normal scores (EPDS ≤ 10 and ASQ: SE below the cut-off).

### Cost-effectiveness analyses

The monetary value of LTP+ was assessed using intention-to-treat analyses over the 6-month trial horizon, from the perspective of health, social care, and the patient family. All analyses were based on complete cases, as missing data were under 2%. Differences in costs and effects between LTP+ added to TAU and TAU-only arms were calculated as the mean difference in cumulative costs and effects over the six-month period of the economic evaluation.

Regression analyses estimated the mean costs and benefits of the LTP+ intervention, adjusting for baseline variables: child and mother age, mother’s years of schooling, total monthly household income, number of family members, housing type, and baseline utility values. These variables are key risk factors for maternal depression and quality of life and have been included in previous clinical efficacy studies and economic evaluations [2, 24, 35, 36]. Total monthly household income, number of family members, and housing type (own or rented) are important determinants of both quality of life and access to healthcare, particularly in lower- and middle-income countries where private healthcare services are often relied upon. As healthcare resource use data were not collected at baseline, including baseline utility values can help adjust for imbalances in quality of life and healthcare use, especially when these are strongly correlated [30].

Both cost and effect regressions were estimated using generalised linear mixed-effects models with cluster-level random intercepts [37]. Given the right-skewed distribution of cost data (mass point near zero, non-negative, and right-tailed, as shown in the additional file, Figure A1), a gamma-distributed mixed-effect model with a log link was applied to estimate adjusted mean costs [24, 37]. For QALYs, which are left-skewed (see additional file, Figure A1), a beta-distributed mixed-effect model with a logit link was used to estimate adjusted mean QALYs [37]. Logistic regressions (Bernoulli-distributed with logit link) were employed for binary indicators of maternal and dyad recovery. All estimations were conducted in STATA 17.0 using the Generalised Structural Equation Modelling (GSEM) command.

In the base-case analyses, uncertainty was addressed by extracting 10,000 nonparametric bootstrapped samples from the data [26]. For each sample, we calculated the incremental costs, incremental effects, and an incremental cost-effectiveness ratio (ICER). The ICER represents the average additional cost of the LTP+ plus TAU arm to achieve one extra effect (QALY or recovery) compared to the TAU-only arm. The ICERs from 10,000 bootstrapped samples were plotted on cost-effectiveness planes, showing cost differences against effect differences between LTP+ plus TAU and TAU-only. The Cost-effectiveness acceptability curves (CEACs) were drawn based on the distribution of the ICERs on the cost-effectiveness planes [38]. CEACs show the likelihood that LTP+ is more cost-effective than TAU-only as a function of the willingness-to-pay (WTP) for one additional QALY or recovery. As there is no established WTP threshold, the amount a society is willing to pay, for a QALY or recovery from depression, we began with a WTP value of zero, increasing it until the likelihood of LTP+ effectiveness reached 100%, following standard economic evaluation practices [23]. The indifference point is conventionally set at a 0.5 probability on the vertical axis [23], above which LTP+ is more likely to be cost-effective compared to TAU-only. The mean ICERs and WTP thresholds yielding a 50% or higher likelihood of LTP+ being cost-effective were compared with Pakistan’s 2015 per capita annual gross domestic product (GDP) and published costs of maternal depression [4, 39, 40].

The base-case analyses of this economic evaluation included only the recurrent or delivery costs of LTP+, the Pakistani tariff rate, and the average unit cost of healthcare use. Sensitivity analyses assessed the robustness of results to: (1) adding a Rs. 300 sessions attendance cost to LTP+ delivery costs, (2) including set-up (non-recurrent) costs (e.g., manual and calendar development, CHW training) alongside delivery costs of LTP+ [27], (3) regressions controlling only for baseline health states, (4) using minimum reported unit costs instead of average unit cost, (5) using maximum reported unit costs, and (6) analysis with at least partial dyad recovery. Partial dyad recovery was defined as one or both mother and child achieving normal scores on the EPDS and ASQ: SE, respectively. These analyses explored the sensitivity of base-case results to variations in the economic evaluation parameters.

The cost-effectiveness analysis was also performed using actual dyad scores on the EPDS and ASQ: SE instead of binary indicators. Dyad scores were calculated by summing the total EPDS and ASQ: SE scores. While recovery thresholds on these scales provide clinically meaningful improvements and simplify analyses, using actual scores offers a more granular approach, capturing all degrees of improvement or worsening in the conditions and their associated costs.

To account for uncertainty, 5,000 nonparametric bootstrapped samples were drawn from the data for each sensitivity analysis to estimate confidence intervals for ICERs. As the evaluation period is only six months, discounting was not applied. All estimations were performed using STATA 17.0.

## Results

### Study participants

In the villages assigned to the LTP+ added to TAU arm, 564 mother-child dyads were recruited, 408 met the inclusion criteria, and 402 completed baseline assessment. In the TAU-only villages, 510 mother-child dyads were recruited, 403 met the eligibility criteria, and 372 completed baseline assessment. The average household size was 9.3 in the LTP+ arm and 9.7 in the TAU-only arm, with monthly household incomes of Rs. 11,648 and Rs. 11,729, respectively. The mean child age was 14.1 months in the LTP+ arm and 13.7 months in the TAU-only arm. The mean child height/weight was 70.2 cm/8.1 kg in the LTP+ arm and 70.0 cm/8.1 kg in the TAU-only arm. For more baseline information, see the published clinical effectiveness paper [14].

Participants in the LTP+ arm attended an average of eight sessions. Of these, 101 (25.1%) attended all sessions, 78 (19.4%) attended nine, 88 (21.8%) attended eight, 57 (14.1%) attended seven, and 33 (8.2%) attended six. Four (3.98%) participants attended three sessions, three (0.75%) did not attend any session, and two (0.50%) attended only one LTP+ session during the trial.

### LTP+ delivery costs

The delivery cost of the LTP+ intervention was Rs. 7,212 ($68.70) per participant, while the per-session cost was Rs. 1,086 ($10.34) based on 399 participants who attended at least one session. Transportation costs for LTP+ delivery, including 20% revisits, were Rs. 3,439 ($32.76) per participant, accounting for 48% of the delivery cost. Revisits were required when scheduled sessions were missed due to logistical issues or participant time constraints (typically, two sessions were delivered per visit). The second major cost component was the wage payment to the therapists and the LTP+ supervisory team for the time of preparation, arrangement, and delivery of LTP+ sessions. This cost amounted to Rs. 1,752 ($16.69) per participant (24% of delivery cost). Printing the LTP+ calendar and manual cost Rs. 1,312 ($12.50) per participant (24% of delivery cost). Rent and babysitting expenses amounted to Rs. 408 ($3.89) per participant (6% of total), while participant compensation of Rs. 300 each totalled Rs. 120,600 ($1,149). A detailed cost breakdown is provided in Table 1.

**Table 1:** The start up and recurrent costs of LTP+ intervention (in 2015 Pakistani rupees*)

Cost heads	Unit cost**	Quantity	Total cost**
**Training and development of CHWs**			
**Set-up (non-recurrent) costs**			
Travel cost	3200 per visit	10 visits	32,000
Project lead wage	240 per hour	80 h	19,231
Senior research psychologist wage	168 per hour	80 h	13,462
Community supervisor/guider wage	48 per hour	80 h	3846
***Manual and calendar development***			
Culturally adapted manual development (English)	6206 per manual	143 manuals	887,458
Culturally adapted manual development (Urdu)	6206 per manual	110 manuals	682,660
Sketches of calendar	500 per calendar	59 calendars	29,500
**Total set-up cost**			1,668,157
**Per participant set-up cost**			4150 (402 participant)
**Recurrent/delivery cost**			
***LTP+ intervention delivery stage***			
Travel cost (transportation)	3200 per visit	360 visits for 720 sessions	1,152,000
Travel cost revisits (transportation)	3200 per visit	72 visits	230,400
***Wage (delivering sessions)***			
Junior research assistant for delivering intervention	120 per hour	540 h	64,904
Senior research psychologist	168 per hour	540 h	90,865
Project lead office and community work	240 per hour	540 h	129,808
Community supervisor/guider	48 per hour	540 h	25,962
***Wage (preparation and travelling for sessions)***			
Junior research assistant for delivering intervention^	120 per hour	743 h	89,303
Senior research psychologist^	168 per hour	743 h	125,024
Project lead office and community work (100%)	240 per hour	743 h	178,606
***Other Resources***			
Rent for acquiring clinic	200 per session	720 sessions	144,000
Babysitter/Aya	50 per participant	402 participants	20,100
***Printing***			
LTP calendar	700 per calendar	750 calendars	525,000
LTP manual	5 per manual	506 manuals	2530
***Monetary incentives***			
Monetary incentive paid to participants	300 per participant	402 participants	120,600
**Total delivery cost**			2,899,102
**Per participant delivery cost**			7212 (402 participant)
**Per participant cost (set-up plus delivery)**			11,361 (402 participant)

Notes: LTP+, Learning Through Play Plus; CHW, community health worker; Rs., Pakistani rupees. *Due to small numbers, all the data are presented in 2015 Pakistani rupees instead of being converted to US dollars (Rs. 105/$1 in 2015). **The unit costs and total costs are rounded to the nearest whole number. ^The junior research assistant and senior research psychologist were community health workers and supervisors

### Missing data

Baseline data were complete for all 774 participants. Three participants dropped out from the LTP+ arm (one migrated and two were not willing to participate), and two dropped out from the TAU-only arm (one was not willing to participate, and the other died) at the 3rd -month follow-up. Three participants dropped out of the intervention arm, and two from the TAU-only arm at the 6th-month follow-up (See Table A2 in the additional file). The missingness indicators for costs and outcomes were not significantly associated with any of the baseline covariates. Thus, all cost-utility analyses (using mother QALYs as the outcome) were conducted on complete cases (*n* = 764). At the 6-month follow-up, EPDS data were missing for two mothers (one from each arm), and ASQ: SE data were missing for four children (three from the LTP+ arm and one from the TAU-only arm). Cost-effectiveness analyses (using mother or dyad recovery as the outcome) were conducted on 762 participants, with children missing ASQ: SE data assigned a recovery value of zero (not recovered), following Husain et al. [35].

### Health and social care costs

The cost of public and private healthcare use is reported in Table A2 in the additional file. The mean per participant inpatient public service cost was Rs. 377 ($3.59) for LTP+ plus TAU and Rs. 276 ($2.63) for TAU-only at the 3rd -month follow-up and increased to Rs. 2705 ($25.76) and to Rs. 4170 ($39.71) at the 6th -month follow-up. This was the largest change among all cost categories during the two follow-ups. Data checks showed that 360 TAU-only and 387 LTP+ participants reported no inpatient healthcare use in the first three months. In the 3–6 month period, only 126 TAU-only and 203 LTP+ participants reported no inpatient use. Notably, ten TAU-only participants reported six or more inpatient episodes during this time.

The mean per participant cost of outpatient clinic visits was Rs. 522 ($4.97) at the 3rd -month follow-up and increased to Rs. 848 ($8.08) at the 6th -month follow-up for the LTP+ arm, while in the TAU-only arm, it increased from Rs. 570 ($5.43) to Rs. 1384 ($13.18) between the two follow-ups. GP clinic visits costed Rs. 369 ($3.51) per person at the 3rd -month and Rs. 39 ($0.37) at the 6th -month follow-up in the LTP+ arm, versus Rs. 301 ($2.87) and Rs. 116 ($1.10) in the TAU-only arm.

Non-hospital costs mainly comprised costs incurred on services from Imams, Hakims, and homeopathic clinics (see the additional file, Table A3). The combined costs of Imams, Hakims, and homeopathic services were Rs. 520 ($4.95) at the 3rd -month follow-up and decreased to Rs. 361 ($3.44) at the 6th -month follow-up in the LTP+ arm. In the TAU-only arm, it decreased from Rs. 488 ($4.65) to Rs. 402 ($3.83) between the two follow-ups. Mothers’ out-of-pocket expenses for availing formal medical services during the three months post-randomisation were Rs. 1036 ($9.87) and Rs. 1885 ($17.95) per participant in the LTP+ plus TAU and TAU-only arms respectively. At the 6th -month follow-up, these costs were Rs. 1273 ($12.12) and Rs. 2049 ($19.51) in the LTP+ plus TAU and TAU-only arms respectively. Mothers were also asked to report out-of-pocket expenses made during visits to doctors for their child healthcare (see additional file, Table A2 for more details). The overall per-dyad costs over six months were Rs. 11,008 ($104.83) in the LTP+ plus TAU arm and Rs. 16,619 ($158.28) in the TAU-only arm.

### Health-related quality of life (HRQol)

**Fig. 1:**
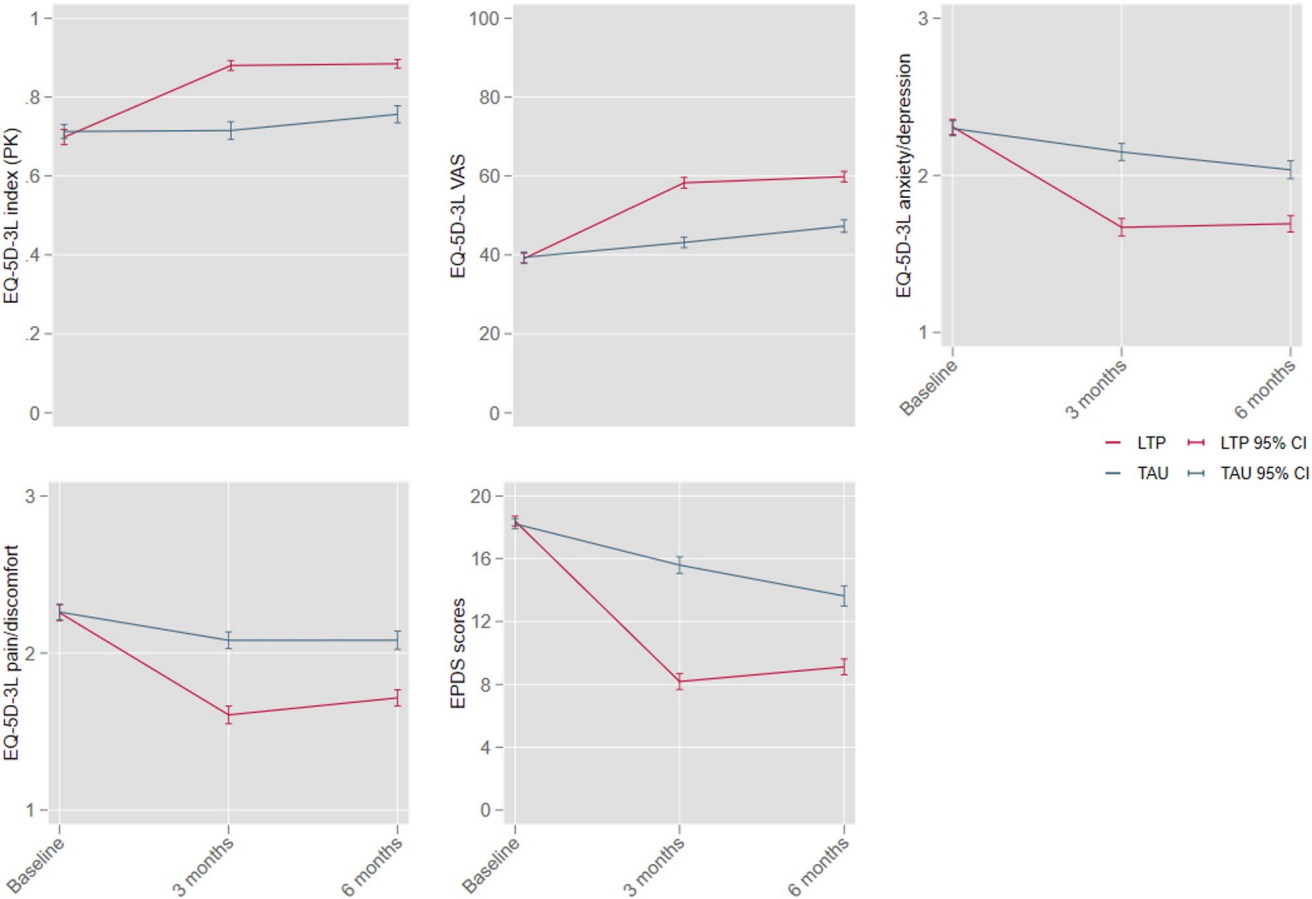
Unadjusted health related quality of life and EPDS scores. EQ-5D-3L, Three-level EuroQol 5-Dimension generic measure of health-related quality of life; PK, Pakistan value set; LTP, learning through play; TAU, treatment as usual; VAS, visual analogue scale; EPDS, Edinburgh postnatal depression scale; CI, Confidence Interval

A significant improvement was observed in maternal quality of life (Fig. 1). Clear improvement was observed in all dimensions of EQ-5D-3L, including anxiety/depression, at the 3rd and 6th month follow-ups in the LTP+ added to TAU arm compared to TAU-only. The difference between the LTP+ and TAU-only arms was higher at the 3rd month (end of intervention) than at the 6th month. At the 6th month follow-up, the difference mainly decreased due to gradual improvement in the scores of the TAU-only arm. Details on each dimension of EQ-5D-3L are given in the additional file (Table A4).

### Mean costs, effects, and ICERs

Table 2 presents the mean and incremental costs (healthcare use and LTP+ delivery) and effects for the LTP+ plus TAU and TAU-only arms, along with cost-effectiveness estimates for the dyad and mother only (detailed regression coefficients are reported in the additional file, Tables A5 and A6). Mean values and 95% confidence intervals were derived from 10,000 non-parametric bootstrap samples [41].

**Fig. 2:**
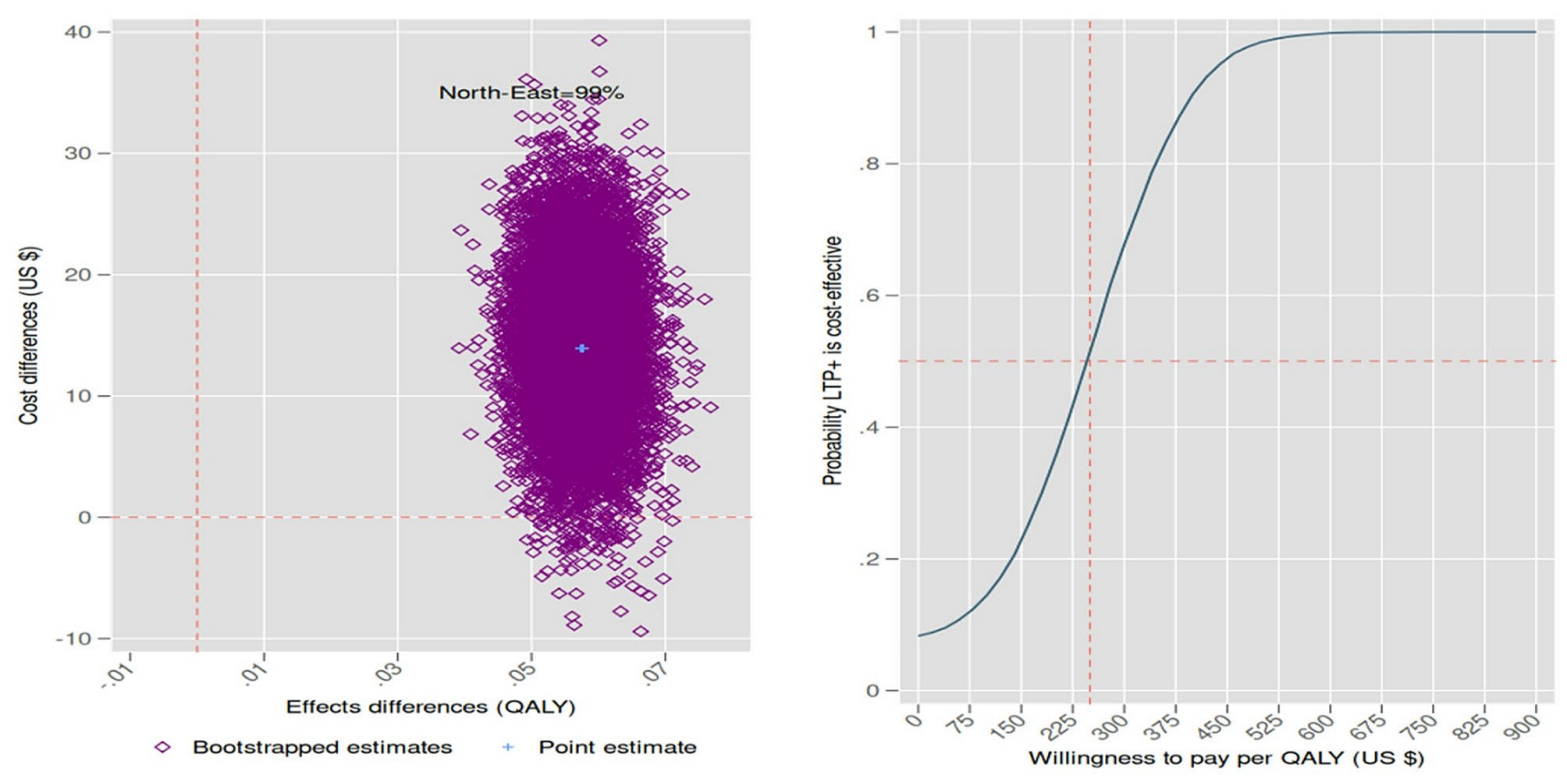
Cost-effectiveness plane and cost-effectiveness acceptability curve (mother QALY, dyad cost)

In partial dyad analyses (as QALYs could not be calculated for children), LTP+ significantly increased dyad costs by $15 (95% CI: $4: $25) and maternal QALY gains by 0.06 (95% CI: 0.05: 0.07). The bootstrapped base-case ICER was $258 (95% CI: $75: $442) per QALY gained, indicating that gaining one additional maternal QALY with LTP+ added to TAU costs an average of $258 when considering its impact on mother and child healthcare use. Approximately 99% of bootstrapped replications fall in the north-east quadrant of the cost-effectiveness plane (panel A, Fig. 2), reflecting higher QALY effects at higher costs. Thus, reliance on the CEAC is essential, showing the probability of LTP+ being cost-effective compared to TAU-only as a function of WTP for one additional QALY (panel B, Fig. 2).

At a hypothetical WTP of $0 per additional QALY, the intervention had a 15% probability of being cost-effective, increasing to 50% at a WTP of $250 (panel B, Fig. 2). The probability of LTP+ being more cost-effective than TAU-only reaches 100% at a WTP of $600. Pakistan’s annual per capita GDP in 2015, according to the IMF, was $1,535, while other sources report figures of $1,421 and $1,380, depending on methodology and data updates.^2^^,^^3^^,^^4^ At the WHO high cost-effectiveness threshold (1x annual per capita GDP), LTP+ appears highly cost-effective. However, several studies have raised concerns about the WHO threshold’s insensitivity to country-specific conditions [39]. A recent study using an opportunity cost approach, with the UK cost-effectiveness threshold as a reference, suggests a cost-effectiveness threshold of 7–53% of Pakistan’s GDP per capita [40]. With a GDP of $1,421, this yields a range of $98–$754. At the midpoint of this range, LTP+ has over a 90% likelihood of being more cost-effective than TAU alone (panel B, Fig. 2).

In the mother-only cost-utility analysis (which, as in Cox et al. [24], excludes any benefits of LTP+ for the child), the incremental costs of LTP+ were $33 (95% CI: $24: $43). The bootstrapped ICER was $582 (95% CI: $404: $769) per QALY gained. All bootstrapped replications fell in the north-east quadrant of the cost-effectiveness plane (additional file, panel A in Figure A2), indicating higher QALY effects at higher costs. The probability of LTP+ being cost-effective reached 50% at a hypothetical WTP of $575. At the upper limit of the opportunity cost-based threshold of $754 (53% of per capita GDP), the likelihood of LTP+ being cost-effective is above 90%. However, it is important to note that the mother-only analysis does not account for the reduced healthcare costs for the child associated with the LTP+ intervention.

**Table 2:** Base case average and incremental costs (2015 US $), effects, and ICER

Unadjusted statistics	Dyad healthcare cost	Intervention cost	Mother QALYs	Participants
Control group (TAU) (mean)	$158	.	0.36	368
Intervention group (LTP+) (mean)	$105	$69	0.42	396
**Adjusted analyses**	Cost (CI)	Effects (CI)	ICER (CI)	Participants
	Dyad Cost	Mother QALY		
Control (TAU)	$153 ($145: $161)	0.36 (0.35: 0.37)	…	368
Intervention (LTP+)	$168 ($165: $178)	0.42 (0.41: 0.42)	…	396
Difference	$15 ($4: $25)	0.060 (0.05:0.07)	$258 ($75: $442) per QALY gained	764
	Mother Cost	Mother QALY		
Control (TAU)	$110 ($103: $117)	0.36 (0.35: 0.37)	…	368
Intervention (LTP+)	$143 ($137: $149)	0.42 (0.41: 0.42)	…	396
Difference	$33 ($24: $43)	0.06 (0.05: 0.07)	$582 ($404: $769) per QALY gained	764
	Dyad Cost	Dyad recovery		
Control (TAU) (11/367)*	$153 ($145: $161)	0.02 (0.01: 0.03)	…	367
Intervention (LTP+) (218/395)*	$168 ($162: $175)	0.55 (0.51: 0.60)	…	395
Difference	$16 ($6: $26)	0.54 (0.49: 0.59)	$29 ($11: $49) per dyed recovered	762
	Mother Cost	Mother recovery		
Control (TAU) (124/367)*	$110 ($103: $117)	0.28 (0.23: 0.33)	…	367
Intervention (LTP+) (261/395)*	$143 ($137: $149)	0.70 (0.65: 0.75)	…	395
Difference	$33 ($24: $42)	0.42 (0.34: 0.50)	$80 ($53: $111) per mother recovered	762

Notes: CI, confidence interval; ICER, incremental cost effectiveness ratio; QALY, quality-adjusted life year; TAU, treatment as usual; LTP+, learning through play plus; US, United States. *The numbers in brackets provide the recovery ratios in the trial arms. 1. Cost includes LTP+ delivery costs, all healthcare use costs of mother and out of pocket healthcare use cost for the child. 2. The 95% CIs are the percentile bootstrap confidence intervals (10,000 replications). 3. Dyad recovery is measured by a binary indicator (equals 1 when mother depression score and child socio-emotional development scores are in normal range, zero otherwise). 4. The adjusted analyses control for baseline health states (or recovery scores), family size and income, mother and child age, mother education, housing type, and include cluster-level random intercepts

**Fig. 3:**
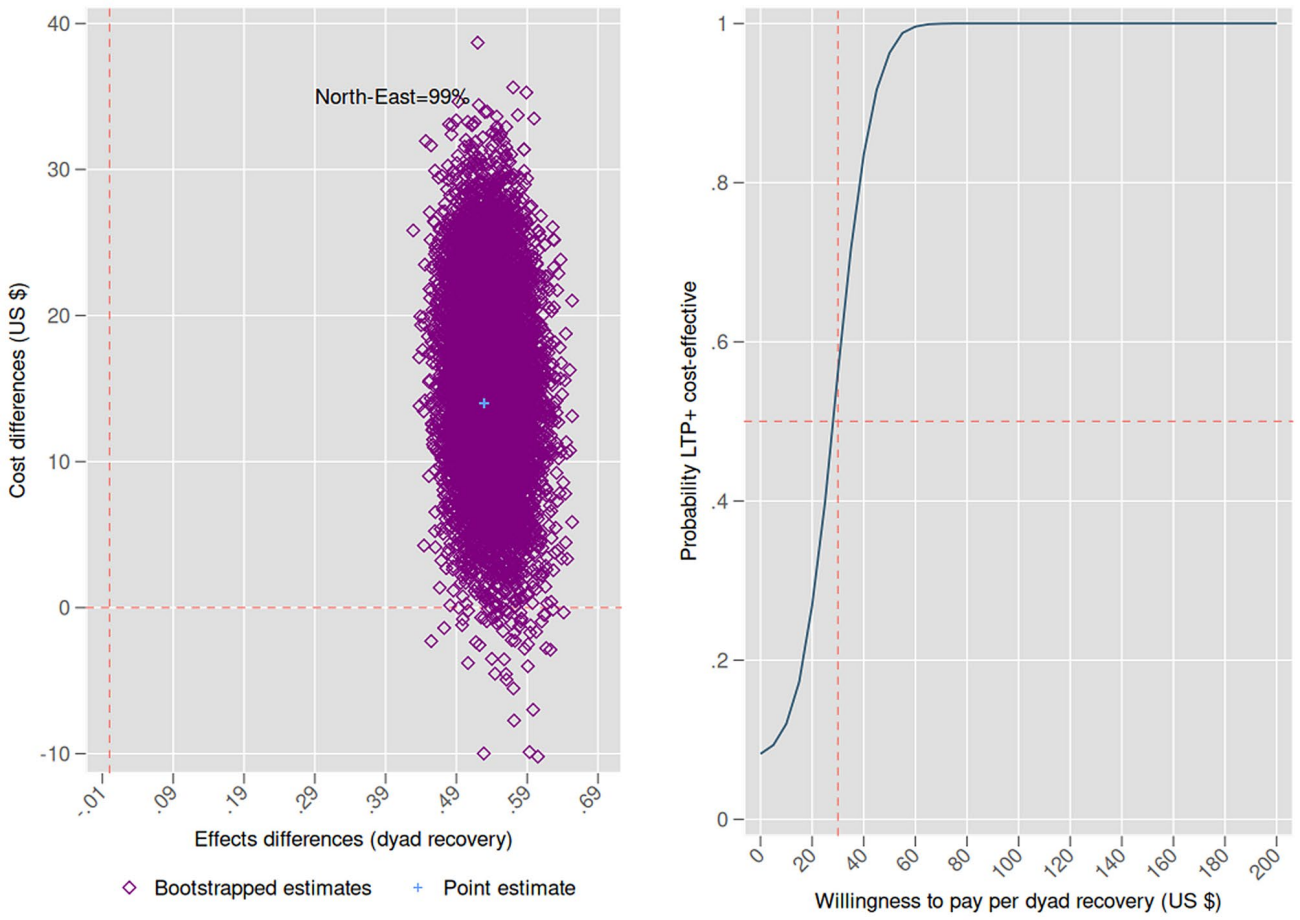
Cost-effectiveness plane and cost-effectiveness acceptability curve (dyad cost and recovery)

Focusing on dyad recovery (cost-effectiveness analysis), LTP+ significantly increased the dyad recovery rate by 0.54 (95% CI: 0.49: 0.59), resulting in an ICER of $29 (95% CI: $11: $49) per dyad recovered. 99% of bootstrapped replications fall in the north-east quadrant of the cost-effectiveness plane (Panel A, Fig. 3), indicating higher recovery rates at higher costs. A WTP of $0 resulted in a 14% probability of LTP+ being cost-effective, while a hypothetical WTP of $60 per dyad recovery yielded a 100% probability of LTP+ being cost-effective (Panel B, Fig. 3).

In the analysis focused on mother-only recovery, LTP+ significantly increased the mother recovery rate by 0.42 (95% CI: 0.34: 0.50) and the cost per mother by $33 (95% CI: $24: $42). The bootstrapped mean ICER was $80 (95% CI: $53: $111) per mother recovered. All bootstrapped replications fall in the north-east quadrant of the cost-effectiveness plane (Additional File: Panel A, Figure A3). A WTP of $0 resulted in a 8% probability of LTP+ being cost-effective, whereas a hypothetical WTP of $1450 per mother recovery showed a 100% probability of LTP+ being cost-effective. The discussion section provides a comparison with existing cost-effectiveness estimates from Pakistan.

### Sensitivity analyses

Table 3 summarises the results of various sensitivity analyses. Adding session attendance costs (Rs. 300 per participant) raised the ICER to $312 (95% CI: $129: $499). Including both set-up (non-recurrent) and delivery (recurrent) LTP+ costs yielded incremental dyad costs of $57 (95% CI: $46: $68) and ICERs of $1,005 (95% CI: $775: $1,261) per maternal QALY gained and $111 (95% CI: $88: $133) per dyad recovered.

Using the maximum reported unit cost instead of the average (see Additional File, Table A1) resulted in no significant difference in dyad costs between LTP+ plus TAU and TAU-only, with LTP+ dominating TAU-only. Conversely, using the minimum unit cost resulted in incremental dyad costs of $32 (95% CI: $25: $38) and an ICER of $562 (95% CI: $441: $717) per maternal QALY gained, highlighting the importance of carefully selecting healthcare resource prices in economic evaluations.

When only baseline health states were used as control variables, the ICER was $257 (95% CI: $79: $441) per QALY gained, closely matching the base-case ICER of $258.

In analyses considering at least partial dyad recovery (where the recovery indicator equals one if either or both dyad members achieve normal scores), the ICER fell from $29 in the base case to $20 (95% CI: $5: $37). Finally, using actual EPDS and ASQ: SE scores, each additional dyad score improvement towards recovery cost less than $1.

**Table 3:** Sensitivity analysis (costs in 2015 US $, all analyses control for baseline characteristics)

Scenario1	Scenario2	Dyad Cost (CI)	Effects (CI)	ICER (CI)	*N*
Outcome measure is same as in Table 2	Blank if not different from Table 2				
Mother QALY	Include Rs. 300 LTP+ attendance costs	$19 ($9: $29)	0.060 (0.05:0.07)	$312 ($129: $499) per QALY gained	764
Mother QALY	Also include set-up cost of LTP+	$57 ($46: $68)	0.06 (0.05: 0.07)	$1,005 ($775: $1,261)	764
Dyad recovery	Also include set-up cost of LTP+	$58 ($48: $68)	0.53 (0.47: 0.57)	$111 ($88: $133)	762
Mother QALY	Use max unit cost from Table A1	$-2 (-$16: $13)	0.06 (0.05: 0.07)	dominant	764
Mother QALY	Use min unit cost from Table A1	$32 ($25: $38)	0.06 (0.05: 0.06)	$562 ($421: $717)	764
Mother QALY	Only baseline health state as control	$15 ($5: $24)	0.06 (0.05: 0.07)	$257 ($79: $441)	764
Outcome measure is different from Table 2					
At least partial dyad recovered		$13 ($3: $24)	0.66 (0.58: 0.73)	$20 ($5: $37)	762
EPDS + ASQ: SE scores		$12 ($1: $23)	-93.98 (-98.03: -89.91)	<$1 ($>0: <$1)	758

Notes: CI, confidence interval; ICER, incremental cost effectiveness ratio; QALY, quality-adjusted life year; EPDS, Edinburgh postnatal depression scale; ASQ: SE, ages and stages questionnaires: social-emotional; US, United States. 1. The 95% CIs are the percentile bootstrap confidence intervals (5,000 replications). 2. Dyad recovery is measured by a binary indicator (equals 1 when mother depression score and child socio-emotional development scores are in normal range, zero otherwise). 3. Partial dyad recovery is measured by a binary indicator (equals 1 when at least one of the dyad member scores are in normal range, zero otherwise). 4. Except one row, all analyses control for baseline health states (or recovery scores), family size and income, mother and child age, mother education, housing type, and include cluster-level random intercepts

## Discussion

This study evaluated the cost-utility and cost-effectiveness of LTP+ added to TAU, an integrated parenting intervention delivered by trained CHWs to address maternal depression and child socio-emotional development in a resource-limited setting. Intervention delivery costs were $68.70 per dyad, primarily driven by transportation and CHW wages. The findings indicate that LTP+ increased total costs but provided health benefits for participants in the treatment arm from a health, social care, and family perspective. The cost per maternal QALY gained was $582 (95% CI: $404: $769) considering only maternal healthcare savings, and $258 (95% CI: $75: $442) when incorporating dyad healthcare cost savings.

Sensitivity analyses showed that the cost per QALY gained was consistently below Pakistan’s 2015 per capita GDP, a threshold deemed highly cost-effective by WHO standards. However, this threshold is considered overly generous for low-income countries, prompting efforts to establish thresholds reflecting the constraints and efficiency of healthcare systems in lower- and middle-income countries [39, 40]. Woods et al. [40] proposed an opportunity cost-based threshold for Pakistan of 7%–53% of per capita GDP ($98–$754 in 2015). The cost per maternal QALY gained from LTP+ fell within this range when healthcare cost savings for both mother and child were included, except when all set-up and delivery costs of LTP+ were considered, resulting in $1,005 (95% CI: $775: $1,261) per QALY. However, set-up costs, such as manual development, are typically excluded from economic evaluations as they are incurred only during the intervention’s initial development stage [27]. Once developed, the manual can be used an indefinite number of times for future patients.

Dyad recovery (normal EPDS and ASQ: SE scores) cost $29 (95% CI: $11: $49), while maternal recovery alone cost $80 (95% CI: $53: $111). Including intervention set-up costs in the sensitivity analysis, LTP+ cost $111 (95% CI: $88: $133) per dyad recovered. The probability of LTP+ being more cost-effective than TAU-only was 100% at a willingness-to-pay of $60 for dyad recovery and $140 for maternal recovery alone.

Most of the existing evidence synthesis about the cost-effectiveness of interventions for postnatal depression is confined to the developed world [16, 42, 43]. The closest lower- and middle-income countries’ studies to ours are the different trials that evaluated the effectiveness of the THP programme in Pakistan and India [11, 17, 18]. The original THP intervention delivered by CHWs was found effective in reducing depression at the 6th and 12th month postnatally, with no effects on the weight and height of children [11]. However, this study did not include any economic evaluation. A peer-delivered THP intervention in Pakistan was found to cost $236 (2015 US dollars) per additional recovery from depression at the 6th month postnatally under the health system perspective and $215 per additional recovery under the societal perspective [17]. The peer-delivered THP was costlier than the LTP+ intervention (which cost $80; CI: $53–$111 per mother recovered at six months from the health system and patient-family perspective), despite being delivered by unpaid volunteer peers. The higher cost per recovery of THP in Sikander et al. [17] seems to result from the lower clinical success of the peer-delivered intervention. Another study evaluated the clinical effectiveness of 18 group-based THP booster sessions (7th to 36th month postnatal) delivered by the same peers to the same mothers [19]. The sessions did not reduce postnatal depression or improve child socio-emotional skills. Maselko et al. [19] found that the competence levels of peers dropped over time and the fidelity of the intervention was not maintained.

In contrast, the peer-delivered THP intervention in India achieved better clinical outcomes than in Pakistan and was cost-effective [18]. The healthcare and societal costs saved completely offset the cost of the intervention in India. The difference in the clinical and cost-effectiveness of LTP+ and peer-delivered THP (or CHWs versus peer-delivered THP) may stem from the mode of delivery or financial incentives. Though the intervention in India was delivered by peers, they were motivated by financial incentives [18]. On the other hand, the peers in Pakistan were altruistically motivated. Altruistic motivation should not involve any fear of loss when the altruist changes their behaviour and may not be made accountable in many instances. That might be one reason why the fidelity in the Pakistani trial fell over time [19]. This argument is strengthened by the success of the original THP trial which was, partly, delivered in the same Kallar Syedan area of Pakistan where the peer-based THP was delivered. Another reason for the lower success of peer-delivered THP in Pakistan compared to India seems to be the geographical context. The Kallar Syedan district in Pakistan is rural with a low literacy rate whereas the Indian trial was conducted in a more urban context where the literacy rate is relatively higher.

LTP+ distinguishes itself from the original THP or the peer-delivered THP by integrating a parental training programme with CBT principles, and hence has significant impacts on different children’s outcomes including improvement in the child’s socio-emotional development [14]. Economic evaluation of trials testing parenting interventions aiming to improve child health are again confined to the developed world [44]. Most of the interventions in developing countries focused on mothers’ nutrition education and tested the impact of such interventions on child cognitive development. A brief review about the cost effectiveness of such interventions is provided in Baek et al. [27]. Very few have tested the role of integrated mental health and parenting interventions in improving both mother and child health outcomes. An RCT in rural Uganda tested an integrated, community-based parenting intervention that targeted both child development (measured by the cognitive composite score according to the Bayley Scales of Infant and Toddler Development (BSID-III)) and maternal wellbeing [45]. Children in the intervention group were found to have significantly higher cognitive scores and mothers reported significantly fewer depressive symptoms. A secondary cost-effectiveness study of the trial found that the ICER for the education intervention in Uganda compared with current practice was $16.50 per cognitive composite score gained and considered the intervention as cost-effective [46]. A CHW-delivered Mentor Mothers programme for antenatal depressed mothers in South Africa, which incorporates cognitive behavioural principles, was found to have no overall effects on the cognitive and motor scale scores on the Bayley Scales of Development [47]. Nevertheless, children in the intervention group were less undernourished, with 10.3% fewer scoring below the 85 threshold on the Bayley Scales for cognitive development.

Early intervention for postnatal depression can prevent chronic depression, improve quality of life, enhance mother-infant bonding, and lead to better cognitive and emotional outcomes in infants [48, 49]. Mothers who recover early are more likely to return to work or home production, show improved productivity, reduced absenteeism, and foster stronger family cohesion and stability [50–52]. As shown in the clinical effectiveness paper from the trial, the LTP+ intervention has significantly improved many aspects of mothers mental and physical health, their parenting competence, and home involvement and organisation skills [14]. High-quality parenting during early childhood is linked to positive mental and physical health outcomes for children in their school years, as well as long-term financial benefits [53, 54].

The LTP+ intervention focused on developing assertiveness skills and enhancing participants’ confidence and communication, as well as reducing social isolation through new relationships and social networks. These new developments could help alleviate the fear of stigma, leading to increased health-care use and reporting [55]. Existing evidence indicates a clear negative association between mental health-related stigma or stigmatising attitudes and help-seeking among individuals [56]. Similarly, group-level anti-stigma interventions have shown at least short-term benefits in combating stigma and discrimination related to mental illnesses [57]. This suggests that the cost of the intervention over a longer period might further decrease.

A key cost in the economic evaluation was LTP+ delivery (£69 per participant) offsetting the healthcare use cost difference ($105 per participant in LTP+ with TAU versus $158 in TAU-only). Real-world implementation may cost less than trial estimates due to economies of scale, such as bulk purchasing and reduced or eliminated transporter profit margins compared to trial delivery (note that 48% of the LTP+ delivery cost was attributed to transportation). Once established, the health system could further save on supervision costs and optimise group sizes during LTP+ sessions.

Real-world implementation and generalisability of the trial results may be affected by several factors. First, participants in the LTP+ group were incentivised with Rs. 300, and the absence of such incentives may impact participant engagement and intervention benefits. Second, about 20% of sessions or assessments were rescheduled due to participant unavailability. In real-world settings, lack of flexibility may hinder adherence and limit treatment benefits. Third, the trial was conducted in a poor, urban area of Pakistan with limited healthcare access. It is unclear if similar benefits would be seen in high-income settings or rural areas. Additionally, certain villages were excluded from randomisation due to concerns about law and order and community reluctance [14], suggesting that sociodemographic and cultural factors may influence the acceptance and benefits of LTP+, limiting the findings’ generalisability.

### Limitations

Resource use data are self-reported, which might introduce recall bias. Baseline healthcare usage costs were not collected. Although we controlled for baseline utility and socioeconomic characteristics, which should mitigate any baseline differences, such an exercise does not guarantee the complete elimination of any bias that might result from baseline differences. Children’s public sector healthcare use and QALY data were not collected, potentially biasing the dyad analysis. If LTP+ reduced children’s public sector healthcare use, as with out-of-pocket expenses, the true cost savings may be underestimated. Medication costs arising from public sector healthcare use were also not included in the analyses. Additionally, the trial’s 6-month time horizon limits assessment of the long-term benefits of LTP+ for mothers and children. Study participants were not blinded to treatment allocation, which could have introduced bias through strategic reporting of costs and health-related quality of life. Furthermore, the geographical proximity of many participants may have allowed them to share their experiences, potentially leading to contamination.

## Conclusion and implications for clinical practice

Significant unmet needs remain among postnatally depressed mothers in lower- and middle-income countries and among ethnic minorities in developed countries due to ill-equipped health systems, poverty, illiteracy, gender inequalities, ethnocultural barriers, and various societal myths. We assessed the cost-effectiveness, relative to the standard of care, of a culturally adapted integrated intervention (LTP+) delivered at participants’ homes or in the nearest health units that can overcome these societal barriers.

Our economic evaluation indicates that LTP+ added to usual care increases both costs and benefits compared to usual care alone. The average cost per maternal QALY gained was $582 when considering only maternal healthcare use and $258 when including the intervention’s impact on both maternal and child healthcare use. The results suggest that the LTP+ intervention for postnatal depression and child socio-emotional development is cost-effective at a willingness-to-pay threshold of 25% or more (approximately ≥ $350) of Pakistan’s 2015 annual per capita GDP per QALY gained, compared to usual care.

Mothers in lower- and middle-income countries and ethnic minority mothers in developed countries often face socio-cultural barriers, preferring home-based treatment over hospital care. LTP+ provides a flexible care model that can be delivered at home or in nearby community or healthcare centres without economic disadvantages. Its delivery by community healthcare workers or trained laypersons can help free up scarce health system resources, particularly in settings with high demand.

## Supplementary Information

Below is the link to the electronic supplementary material.

Supplementary Material 1

## Data Availability

The datasets used and/or analysed during the current study are available from the corresponding author on reasonable request.

## Footnotes

1 The age-specific ASQ: SE cutoffs used were as follows: ≤6 months: 45, > 6 to ≤ 12 months: 48, > 12 to ≤ 24 months: 50, > 24 to ≤ 30 months: 57, and > 30 to ≤ 36 months: 59. For details, see: ASQSE.pdf.

2
World Economic Outlook (October 2024) - GDP per capita, current prices.

3
World Development Indicators | DataBank.

4
Pakistan GDP per capita.

## Abbreviations

CBT: Cognitive behavioural therapy
CEACs: Cost-effectiveness acceptability curves
CHWs: Community health workers
EPDS: Edinburgh Postnatal Depression Scale
GDP: Gross Domestic Product
ICER: Incremental cost-effectiveness ratio
LTP+: Learning Through Play Plus
QALYs: Quality adjusted life years
TAU: Treatment as usual
THP: Thinking Healthy Programme
UK: United Kingdom
US: United States
$: US dollar
Rs: Pakistani rupees
