# Supplementary material for: Economic evaluation of Learning Through Play Plus in comparison to usual care for depressed mothers alongside a randomised controlled trial
Source: BMC Health Serv Res. 2026 Feb 4;26:328. doi: 10.1186/s12913-026-14113-0 (PMC12958606; doi:10.1186/s12913-026-14113-0)

## Table A1: Healthcare Service Unit Costs (2015 Pakistani rupees)

|  | Unit of measure | Cost range | unit cost |
| --- | --- | --- | --- |
| Hospital costs | | | |
| Psychiatric inpatient | Day | 300-1500 | 900 |
| Physical inpatient | Day | 150-5000 | 2575 |
| Accident and Emergency | Attendance | 500-10000 | 5250 |
| Psychiatric/psychologist outpatient | Attendance | 300-1000 | 650 |
| Physical outpatient | Attendance | 200-1000 | 600 |
| GP physical | Attendance | 100-500 | 300 |
| GP psychological | Attendance | 300-400 | 350 |
| Non-hospital costs | | | |
| Imam/maulvi | Attendance | 50-500 | 275 |
| Pir/Faqeer | Attendance | 1000-5000 | 3000 |
| Hakim | Attendance | 200-2500 | 1350 |
| Homeopathic | Attendance | 200-2500 | 1350 |
| Volunteer agency | Attendance | 50-100 | 75 |
| Social services | Attendance | 0-1000 | 500 |

*Note: For each category, the hospital and non-hospital costs were collected from four hospitals/non-hospital services operating in the catchment area and average out to arrive at the unit cost.*

## Table A2: Mean and total costs of healthcare resources (in 2015 Pakistani rupees)

|  | LTP+ |  |  | TAU |  |  |
| --- | --- | --- | --- | --- | --- | --- |
|  | Mean | SD | Participants | Mean | SD | Participants |
| Baseline to 3^rd^ Month Follow up |  |  |  |  |  |  |
| Inpatient (physical) | 377 | 2560 | 399 | 276 | 1689 | 370 |
| Outpatient clinic (physical) | 522 | 1026 | 399 | 570 | 1039 | 370 |
| GP (physical) | 369 | 727 | 399 | 301 | 582 | 370 |
| Any other doctor (physical) | 31 | 287 | 399 | 3 | 40 | 370 |
| Any other primary care (physical) | 42 | 233 | 399 | 44 | 211 | 370 |
| Any other primary care (psychological) | 0 | 0 | 399 | 0 | 0 | 370 |
| Faith healers/homeopathic | 520 | 1022 | 399 | 488 | 974 | 370 |
| Mother total (health & social care sector) | 1863 | 3176 | 399 | 1686 | 2320 | 370 |
| Mother own medical expenses | 1036 | 2287 | 399 | 1885 | 2377 | 370 |
| Child cost | 1276 | 1756 | 399 | 2422 | 2989 | 370 |
| Dyad total (0 to 3 months) | 4173 | 4613 | 399 | 6008 | 4948 | 370 |
| 4^th^ to 6^th^ Month follow up |  |  |  |  |  |  |
| Inpatient (physical) | 2705 | 3611 | 396 | 4170 | 4519 | 368 |
| Outpatient clinic (physical) | 848 | 988 | 396 | 1384 | 1325 | 368 |
| GP (physical) | 39 | 184 | 396 | 116 | 358 | 368 |
| Any other doctor (physical) | 9 | 100 | 396 | 4 | 57 | 368 |
| Any other primary care (physical) | 16 | 96 | 396 | 66 | 181 | 368 |
| Any other primary care (psychological) | 15 | 92 | 396 | 23 | 111 | 368 |
| Faith healers/homeopathic | 361 | 848 | 396 | 402 | 799 | 368 |
| Mother total (health & social care sector) | 3995 | 4578 | 396 | 6169 | 5512 | 368 |
| Mother own medical expenses | 1273 | 2398 | 396 | 2049 | 2532 | 368 |
| Child cost | 1551 | 2324 | 396 | 2408 | 2177 | 368 |
| Dyad total (4 to 6 months) | 6819 | 6848 | 396 | 10625 | 7752 | 368 |
| Dyad grand total (0 to 6 months) | 11008 | 9419 | 396 | 16619 | 9815 | 368 |
| Per session LTP (recurrent) | 1115 | 1530 | 399 | . | . | . |
| Per participant LTP (recurrent) | 7412 | 2496 | 402 | . | . | . |

*Notes: LTP+, Learning Through Play Plus; TAU, Treatment As Usual; SD, standard deviation.*

## Table A3: Mean health and social care resource use

|  | **LTP+** | | |  | **TAU** | | |
| --- | --- | --- | --- | --- | --- | --- | --- |
|  | Mean | SD | Participants |  | Mean | SD | Participants |
| **Baseline to 3^rd^ Month Follow up** |  |  |  |  |  |  |  |
| Inpatient (physical) | 0.15 | 0.99 | 399 |  | 0.11 | 0.66 | 370 |
| Outpatient clinic (physical) | 0.87 | 1.71 | 399 |  | 0.95 | 1.73 | 370 |
| GP (physical) | 1.23 | 2.43 | 399 |  | 1.01 | 1.94 | 370 |
| Any other doctor (physical) | 0.07 | 0.64 | 399 |  | 0.01 | 0.09 | 370 |
| Any other primary care (physical) | 0.08 | 0.47 | 399 |  | 0.09 | 0.42 | 370 |
| Imam/Maulvi | 0.31 | 0.46 | 399 |  | 0.26 | 0.45 | 370 |
| Pir/Faqeer | 0.13 | 0.34 | 399 |  | 0.12 | 0.32 | 370 |
| Homeopathic | 0.04 | 0.20 | 399 |  | 0.05 | 0.23 | 370 |
| **4^th^ to 6^th^ Month follow up** |  |  |  |  |  |  |  |
| Inpatient (physical) | 1.05 | 1.40 | 396 |  | 1.62 | 1.76 | 368 |
| Outpatient clinic (physical) | 1.41 | 1.65 | 396 |  | 2.31 | 2.21 | 368 |
| GP (physical) | 0.13 | 0.61 | 396 |  | 0.39 | 1.19 | 368 |
| Any other doctor (physical) | 0.02 | 0.22 | 396 |  | 0.01 | 0.13 | 368 |
| Any other primary care (physical) | 0.03 | 0.19 | 396 |  | 0.13 | 0.36 | 368 |
| Any other primary care (psychological) | 0.03 | 0.19 | 396 |  | 0.05 | 0.22 | 368 |
| Imam/Maulvi | 0.22 | 0.41 | 396 |  | 0.47 | 0.50 | 368 |
| Pir/Faqeer | 0.11 | 0.31 | 396 |  | 0.11 | 0.32 | 368 |
| Homeopathic | 0.02 | 0.13 | 396 |  | 0.02 | 0.14 | 368 |
| *Notes: LTP+, Learning Through Play Plus; TAU, Treatment As Usual; SD, standard deviation* | | | | | | | |

## Table A4: Health-Related Quality of Life

|  |  | LTP+ |  |  | TAU |  |  |
| --- | --- | --- | --- | --- | --- | --- | --- |
| Dimensions (n, %) |  | Baseline | 3^rd^ Month | 6^th^ Month | Baseline | 3^rd^ Month | 6^th^ Month |
| Mobility | 1 | 63  (15.7%) | 220  (54.7%) | 231  (57.4%) | 70  (18.8%) | 66  (17.7%) | 123  (33.0%) |
|  | 2 | 326  (81.0%) | 173  (43.0%) | 161  (40.0%) | 293  (78.7%) | 283  (76.0%) | 230  (61.8%) |
|  | 3 | 13  (3.2%) | 6  (1.4%) | 4  (0.99%) | 9  (2.41%) | 21  (5.64%) | 15  (4.03%) |
| Selfcare | 1 | 184  (45.7%) | 301  (74.8%) | 299  (74.3%) | 170  (45.6%) | 144  (38.7%) | 187  (50.2%) |
|  | 2 | 207  (51.4%) | 93  (23.1%) | 93  (23.1%) | 196  (52.6%) | 193  (51.8%) | 163  (43.8%) |
|  | 3 | 11  (2.7%) | 5  (1.24%) | 4  (0.99%) | 6  (1.61%) | 33  (8.87%) | 18  (4.83%) |
| Usual Activities | 1 | 40  (9.9%) | 220  (54.7%) | 226  (56.2%) | 45  (12.0%) | 79  (21.2%) | 117  (31.4%) |
|  | 2 | 340  (84.5%) | 175  (43.5%) | 167  (41.5%) | 315  (84.6%) | 259  (69.6%) | 228  (61.2%) |
|  | 3 | 22  (5.4%) | 4  (0.99%) | 3  (0.74%) | 12  (3.22%) | 32  (8.60%) | 23  (6.18%) |
| Pain/Discomfort | 1 | 14  (3.4%) | 176  (43.7%) | 125  (31.0%) | 10  (2.68%) | 37  (9.94%) | 45  (12.0%) |
|  | 2 | 271  (67.4%) | 204  (50.7%) | 259  (64.4%) | 255  (68.5%) | 266  (71.5%) | 248  (66.6%) |
|  | 3 | 117  (29.1%) | 19  (4.7%) | 12  (2.98%) | 107  (28.7%) | 67  (18.0%) | 75  (20.1%) |
| Anxiety/Depression | 1 | 6  (1.4%) | 153  (38.0%) | 136  (33.8%) | 2  (0.53%) | 29  (7.79%) | 51  (13.7%) |
|  | 2 | 266  (66.1%) | 225  (55.9%) | 246  (61.1%) | 257  (69.0%) | 257  (69.0%) | 253  (68.0%) |
|  | 3 | 130  (32.3%) | 21  (5.2%) | 14  (3.4%) | 113  (30.3%) | 84  (22.5%) | 64  (17.2%) |

*Notes: LTP+, Learning Through Play Plus; TAU, Treatment As Usual.*

| Table A5: Coefficients of the base-case dyad costs and maternal QALYs regressions | | | | | | |
| --- | --- | --- | --- | --- | --- | --- |
|  | Cost (US $) | Lower CI | Upper CI | QALYs | Lower CI | Upper CI |
| LTP+ | 0.100* | -0.003 | 0.203 | 0.248*** | 0.209 | 0.288 |
| QALYs (baseline) | -0.133** | -0.245 | -0.02 | 0.374*** | 0.321 | 0.428 |
| Child age (baseline) | -0.003 | -0.008 | 0.002 | 0.001 | -0.001 | 0.003 |
| Mother age (baseline) | -0.005 | -0.012 | 0.001 | -0.002 | -0.005 | 0.001 |
| Household monthly income (baseline, 000s $) | -0.381** | -0.744 | -0.017 | 0.058 | -0.109 | 0.226 |
| Mother education in years (baseline) | -0.006 | -0.016 | 0.004 | 0.004* | -0.001 | 0.009 |
| Own house | 0.119* | -0.018 | 0.256 | 0.077** | 0.016 | 0.137 |
| Total family members | 0.008** | 0 | 0.015 | 0 | -0.003 | 0.003 |
| Constant | 5.163*** | 4.912 | 5.414 | -0.737*** | -0.852 | -0.622 |
| Observations | 764 |  |  |  |  |  |
| Notes: * p<0.1, ** p<0.05, *** p<0.01. *CI, confidence interval; QALY, quality-adjusted life year; LTP+, Learning Through Play Plus.*  *1. Includes random village intercepts.*  *2. Cost equation estimated with gamma distribution and log link.*  3. QALYs equation estimated with beta distribution and logit link.  4. Base category for the house status is rented housing. | | | | | | |

| Table A6: Coefficients of the base-case dyad costs and dyad recovery regressions | | | | | | |
| --- | --- | --- | --- | --- | --- | --- |
|  | Cost (US $) | Lower CI | Upper CI | QALYs | Lower CI | Upper CI |
| LTP+ | 0.103** | 0.002 | 0.205 | 4.103*** | 3.321 | 4.886 |
| EPDS score (baseline) | 0.007 | -0.006 | 0.019 | -0.081** | -0.152 | -0.011 |
| Child age (baseline) | -0.002 | -0.007 | 0.002 | 0.001 | -0.024 | 0.027 |
| Mother age (baseline) | -0.006 | -0.012 | 0.001 | -0.009 | -0.048 | 0.03 |
| Household monthly income (baseline, 000s $) | -0.377** | -0.752 | -0.001 | 0.928 | -1.32 | 3.177 |
| Mother education in years (baseline) | -0.008 | -0.018 | 0.002 | 0.029 | -0.028 | 0.086 |
| Own house | 0.118* | -0.018 | 0.254 | -0.282 | -1.122 | 0.558 |
| Total family members | 0.007* | -0.001 | 0.014 | 0.038* | -0.006 | 0.082 |
| Constant | 5.002*** | 4.668 | 5.336 | -2.460** | -4.458 | -0.463 |
| Observations | 762 |  |  |  |  |  |
| Notes: * p<0.1, ** p<0.05, *** p<0.01. *CI, confidence interval; EPDS, Edinburgh postnatal Depression scale; LTP+, Learning Through Play Plus.*  *1.Includes random village intercepts.*  *2. Cost equation estimated with gamma distribution and log link.*  3. Dyad (binary indicator) equation estimated with Bernoulli distribution and logit link.  4. Base category for the house status is rented housing. | | | | | | |

## Figure A1: Density plots of total costs (2015 US $) and quality adjusted life years


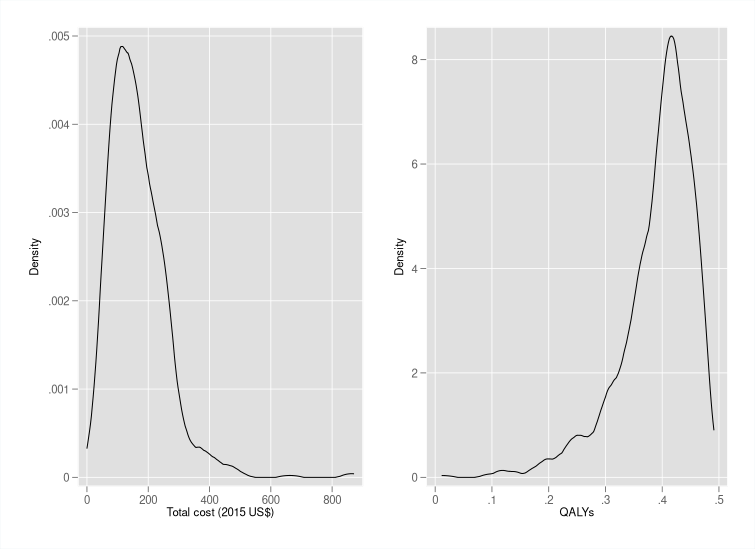


## Figure A2: cost-effectiveness plane and acceptability curves (mother cost and QALYs)


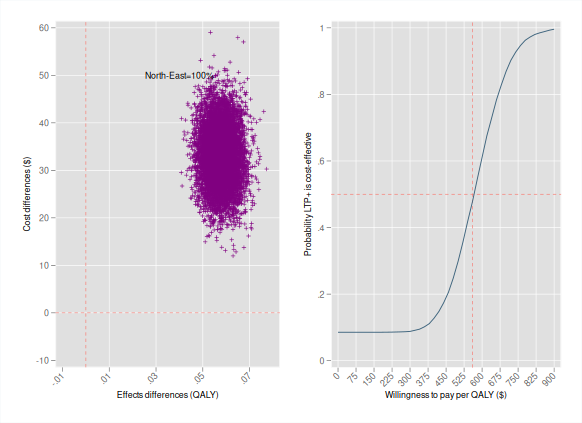


## Figure A3: cost-effectiveness plane and acceptability curves (mother cost and recovery)


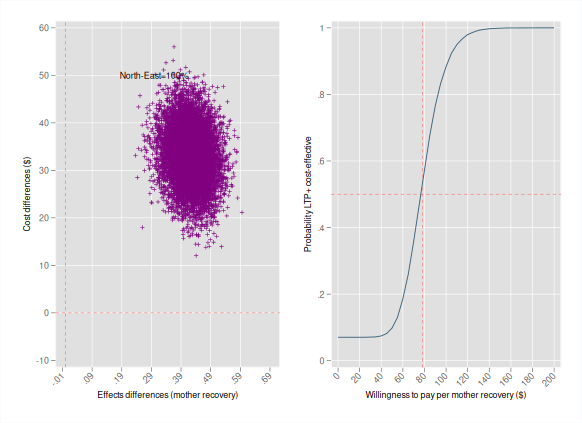

Supplement: Supplementary file 1 — Supplementary Material 1 [file 12913_2026_14113_MOESM1_ESM.docx]
